# Applying Photovoice to uncover fundamental causes of health inequality in local health contexts

**DOI:** 10.3389/fpubh.2026.1737022

**Published:** 2026-03-19

**Authors:** Marisa Booty, Madelyne Culbertson, Mason Taylor, Dakota Heise, Heather Stone, Katherine Leanne Kommer, Jennifer Gulley, Jeanette Hart, Christina Nentwick, Margaret McGladrey

**Affiliations:** 1 Department of Sociology, University of Kentucky College of Arts and Sciences, Lexington, KY, United States; 2 Center for Public Health Systems and Services Research, University of Kentucky College of Public Health, Lexington, KY, United States; 3 Markey Cancer Center, University of Kentucky, Lexington, KY, United States; 4 Montgomery County Health Department, Mt. Sterling, KY, United States; 5 Division of Prevention and Quality Improvement, Kentucky Department for Public Health, Frankfort, KY, United States; 6 Oldham County Health Department, La Grange, KY, United States; 7 Fleming County Health Department, Flemingsburg, KY, United States; 8 Lexington-Fayette County Health Department, Lexington, KY, United States; 9 Department of Health Management and Policy, University of Kentucky College of Public Health, Lexington, KY, United States

**Keywords:** community health assessment, fundamental cause theory, participatory action research, Photovoice, social-ecological model, structural determinants of health

## Abstract

**Introduction:**

Participatory action research methods like Photovoice are uniquely equipped to identify structural determinants of health based on lived experiences of how they influence proximal and distal population health outcomes. This study uses Photovoice data to examine how structural determinants impact community health needs.

**Methods:**

Qualitative thematic analysis used focus group discussion data (25 transcripts) from four Kentucky counties that embedded Photovoice into their local health department community health assessment processes. Coded excerpts were examined for ways in which participants' community health concerns reflected structural issues rather than individual health behaviors.

**Results:**

Participants perceived issues such as houselessness, food insecurity, and transportation as resulting from structural factors beyond the scope of local public health departments. Rather than individual interventions to improve health, participants often discussed the need for system changes in health promotion. The findings show that while local organizations can contribute to addressing health disparities, violence prevention and health promotion require larger policy decisions to reduce health disparities.

**Conclusion:**

Highlighting the roots of health disparity as lived and understood by community members through Photovoice may indicate specific areas where cross-sector engagement and government intervention is most critical to improve community health outcomes.

## 1 Introduction

Photovoice is a participatory action research method that has been identified as a way to engage community stakeholders with various roles (e.g., advocates, practitioners, policymakers) in community assessment processes (1). The goal of Photovoice is to use photography to document participants' lived experiences and facilitate group discussions that increase collective consciousness related to community priorities and promote action toward social change (1, 2). The participatory ideals of Photovoice allow individuals who may not have a seat at the decision-making table to collaborate and vocalize their priorities for bolstering community strengths and addressing local issues. Strack et al. (1) outlined four primary categories in which Photovoice is utilized: (1) individual-level intervention (“photovention”) to reduce stigma and increase self-efficacy; (2) community assessment; (3) community capacity building; (4) advocacy for change. Related to the use of Photovoice for community assessment, several prior studies have used Photovoice to assess community priorities in addressing physical activity programming (3), adolescent substance use (4), and local environmental health hazards (5). The use of photography can provide contextualized information about tangible avenues for addressing community needs, such as photos of dumpsters placed next to playgrounds and factory smog that illustrate the lived experience of environmental health hazards (5).

The participatory goals and assessment capabilities of Photovoice thus align well with those of local health departments, particularly in facilitating the community health assessment (CHA) process. The CHA involves collecting primary data in collaboration with community stakeholders to inform the prioritization of health department policies, strategies, and interventions during the subsequent community health improvement planning (CHIP) phase. Implementation of Photovoice within the CHA process thus improves the method's limitation in reaching policymakers to influence decision-making (6).

In Kentucky, Photovoice has been embedded in several county CHA processes, using the social-ecological model (SEM) as an analytical framework (7). The SEM typically positions an individual's health outcomes at the center of nested circles, with the most direct influences (e.g., individual skills, knowledge and attitudes, interpersonal relationships) situated within the inner circles and more indirect influences (e.g., community partnerships, cultural norms, public policy) within the outer circles (8–11). The SEM is a valuable tool for guiding public health interventions because it illustrates the ways in which health risks and decision-making are constrained by both individual-level beliefs and behaviors as well as structural factors to inform various targets for intervention (8). The outer circles of the SEM in particular highlight social and structural determinants of health and provide a framework for understanding the underlying mechanisms (e.g., social, political, economic) that create inequitable conditions and influence population health (12). Following fundamental cause theory (13), the inequitable distribution of power, prestige, money, knowledge, and beneficial social connections due to structural determinants can be leveraged to ensure better health outcomes for individuals and groups with advantageous circumstances through greater access to protective factors and less exposure to risk factors. Rather than highlighting individual and interpersonal avenues for changing behaviors to improve population health outcomes, fundamental cause theory suggests the need to investigate and dismantle structural factors, systems, and patterns of inequitable distribution of socioeconomic resources that reproduce disparity over time. Thus, the SEM has been adopted by leading health agencies, including the Centers for Disease Control and Prevention and the World Health Organization, to examine various public health concerns (8, 9, 14).

The purpose of this study is to use participatory Photovoice discussion data to demonstrate how community members' health priorities in four Kentucky counties reflect structural issues that may fall outside local health departments' scope of practice. Participatory action research methods like Photovoice are uniquely equipped to identify structural determinants based on lived experiences of how they influence proximal and distal population health outcomes. Prior research has shown how Photovoice photo-elicitation and focus group discussions can meaningfully inform local health departments' intervention efforts to align with community member priorities (6). While interventions targeting social determinants of health may mitigate the symptoms of inequity, such as food insecurity or housing instability, they do not resolve systemic bias and hierarchical power dynamics. Structural, environmental, and systems transformations are beyond the traditional clinical scope of local health departments and instead require cross-sector engagement among health organizations and non-health partners such as local policymakers and chambers of commerce. The need for cross-sector collaboration beyond healthcare entities is support by the Public Health 3.0 model, which prioritizes structural issues to address community health disparities beyond traditional public health venues such as clinics and health departments (15). Highlighting the roots of health disparities as they are lived and understood by community members through this study may thus forward the aims of Public Health 3.0 by indicating specific areas where cross-sector engagement is most critical.

## 2 Materials and methods

### 2.1 Data collection

This study includes focus group discussion data from Photovoice projects conducted between Fall 2022 and Spring 2025 in four Kentucky counties. Local health departments initiated the Photovoice projects, starting with the accreditation coordinator for County #1 (Author 8) who had worked with the first and senior author on another Photovoice project and had wanted to integrate Photovoice into their community health assessment work. The first, senior, and eighth author presented the project at their annual state public health association conference, which led to interest from other local health departments; the state health department also shared information about Photovoice partnership opportunities through their quality improvement listserv. All local health departments used convenience sampling to recruit CHA participants through listservs, coalitions, and local health fairs, and participants came from various backgrounds in community health such as health department employees, non-profit professionals, and engaged citizens. The health departments had different project timelines, some with more time allocated to recruitment and others with more aggressive timelines, that yielded varied numbers of participants. Compared with normal population distributions, female-identifying participants were over-represented in the sample; however, this is not unexpected given that the majority of U.S. healthcare and social work professionals are female (16, 17). Compared with U.S. Census data, the participants in County 1 and County 2 Photovoice projects are more racially diverse, while participants in the small rural Counties 3 and 4 were racially homogenous; however, the percentage of Black/African-American residents of these counties are only 4.1 and 2.5%, respectively (18). Consistent with U.S. Census data for these counties, educational backgrounds varied widely, with representation of people with high school degrees through graduate education (18).

Four Photovoice sessions were conducted for each group using a similar protocol to the HEALing Communities Study Photovoice project (7). Photovoice projects began with an orientation session during which the facilitator led participants through the nominal group technique, where participants were given time to brainstorm and list potential topics for photography related to community health strengths. Example topics generated by participants included “*addressing houselessness and meeting housing needs*”, “*harm reduction and recovery supports*”, “*rural areas of the county don't have resources*”, and “*access to healthy foods*”, with significant overlap in topics across groups. Participants then shared their photo topics with the group, and a comprehensive list of the participant-generated topics on community health strengths was recorded; this process was repeated to generate a list of potential photo topic ideas on community health concerns. Participants could select any topic from the lists to photograph and present during the respective strengths and concerns group discussions.

During the photo discussion session on community strengths, participants shared their photos with the group and the facilitator used the SHOWeD process to guide group discussion about the photos. The SHOWeD questions, developed by the founders of the Photovoice method, include: (1) what do you **S**ee here (2) what is really **H**appening here (3) how does this relate to **O**ur lives (4) Why does this condition exist and (5) what can we **D**o about it (19). Each session included the facilitator and an analyst from the research team who took notes during the group discussions to reflect back at participants in the following session as a member-check strategy. When the analyst's notes were shared with participants, participants were given the opportunity to comment on whether the analyst's summaries adequately reflected participant perspectives and to suggest changes if needed. This photo discussion process was repeated during the subsequent session on community health challenges. The final Photovoice session involved a collaborative analysis of photos and session notes in which participants created summative captions to accompany all photos taken by their group, and the projects ended with discussions of dissemination and potential next steps.

For each project, the local health department “sponsor” (Authors 5, 7, 8, 9, and 10) worked with the senior author to develop a contract and scope of work in which the sponsors facilitated recruitment through their standard channels, explained what community health assessments are and their health departments' goal for Photovoice integration during the orientation session (e.g., amplifying community voice, supplementing survey data with rich visual narratives), and combined their notes on photo discussions with those of the university team between sessions. The sponsors also led dissemination activities chosen by participants (e.g., exhibiting and presenting at community health forums for health improvement planning, creating displays at local libraries, distributing postcards at health fairs). The university team used their published Photovoice protocol to create customized PowerPoint slides and facilitate sessions, lead notetaking, and collect evaluation data as well as completed secondary thematic analysis of findings across projects. With participants' permission, the research team used a Zoom business account to assign participant ID numbers and record and transcribe each 90-min session. All transcripts were reviewed to correct typographical errors and remove identifying information.

The study received a Not Human Subjects Determination from the Institutional Review Board at the senior author's institution, as it was classified as a quality improvement project and thus exempt from further oversight.

### 2.2 Thematic analysis

The researchers analyzed 25 transcripts (12 from County #1, 9 from County #2, 2 from County #3, 2 from County #4) using thematic analysis to examine how the participants' prioritized community health issues were related to fundamental causes of disease. The codebook for this study drew from the community and policy levels of the codebook developed by Balvanz et al. (20) due to the aligned scopes and project methodologies. Community-level codes included: *stereotypes, contextual facilitators and barriers to health and wellness, cross-system coordination*, and *workforce issues*. Policy-level codes included: *structural discrimination and power; status loss and labeling; policies needed to promote health and wellness;* and *fundamental causes of disease*. Additionally, the codebook includes *barriers* and *facilitators* codes to indicate community- and policy-level factors that act as barriers to or facilitators of community health. Once the researchers obtained consensus on the definitions and interpretations of the codebook, the codes were applied to each transcript from the orientation, strengths, and challenges focus group discussion sessions for the four counties included in this study.

During the thematic analysis, we examined excerpts coded to *fundamental causes of disease* and *barriers*. Participant concerns had significant overlap with the five core domains prioritized by the Centers for Medicare & Medicaid Accountable Health Communities Health-Related Social Needs screening tool (homelessness, food insecurity, transportation difficulties, utility assistance needs, and interpersonal safety). The Centers for Medicare & Medicaid now requires healthcare settings to screen patients for these five domains and calls for changes beyond individual and interpersonal intervention to address disparities within these domains. Thus, the researchers found the five core domains to be an appropriate way to organize the structural issues voiced by Photovoice participants through the CHA processes.

## 3 Results

Table 1 provides demographic information for the Photovoice participants included in this study. County population totals from the U.S. Census Bureau (18) and rural-urban classifications based on the U.S. Department of Agriculture rural-urban commuting area codes (21) are also included in the table. County #2 did not collect information on participants' education level due to partner organization preference.

**Table 1:** Demographic characteristics of participants by county.

**Item**	**Total (*n* = 65) % (*n*)**	**County #1 (*n* = 23) % (*n*)**	**County #2 (*n* = 29) % (*n*)**	**County #3 (*n* = 4) % (*n*)**	**County #4 (*n* = 9) % (*n*)**
**County characteristics**					
Population size	–	36,972	322,570	28,114	67,607
Classification	–	Rural	Urban	Rural	Urban
**Age in years** ^a^					
18–30	13.8 (9)	–	24.1 (7)	25.0 (1)	11.1 (1)
31–45	16.9 (11)	–	27.6 (8)	25.0 (1)	22.2 (2)
46–60	20.0 (13)	–	24.1 (7)	50.0 (2)	44.4 (4)
61–75	1.5 (1)	–	3.4 (1)	0.0	0.0
76 +	0.0	–	0.0	0.0	0.0
Unknown	47.7 (31)	100 (23)	20.7 (6)	0.0	22.2 (2)
**Sex**					
Male	27.7 (18)	30.5 (7)	27.6 (8)	0.0	33.3 (3)
Female	63.1 (41)	69.5 (16)	55.2 (16)	100.0 (4)	55.6 (5)
Unknown	9.2 (6)	0.0	17.2 (5)	0.0	11.1 (1)
**Race/ethnicity** ^b^					
White	78.5 (51)	82.6 (19)	69.0 (20)	100.0 (4)	88.9 (8)
Black	9.2 (6)	17.3 (4)	6.9 (2)	0.0	0.0
Asian	1.5 (1)	4.3 (1)	0.0	0.0	0.0
Hispanic	4.6 (3)	4.3 (1)	3.4 (1)	0.0	11.1 (1)
Other or N/A	3.1 (2)	0.0	6.9 (2)	0.0	0.0
Unknown	9.2 (6)	0.0	17.2 (5)	0.0	11.1 (1)
**Education** ^c^					
High school degree or equivalent	9.2 (6)	22.0 (5)	–	0.0	11.1 (1)
Some college, no degree	9.2 (6)	22.0 (5)	–	25.0 (1)	0.0
Associate degree	3.1 (2)	8.7 (2)	–	0.0	0.0
Bachelor's degree	20.0 (13)	30.5 (7)	–	75.0 (4)	22.2 (2)
Graduate education	12.3 (8)	13.0 (3)	–	0.0	55.6 (5)
Unknown	46.2 (30)	0.0	100 (29)	0.0	11.1 (1)

^a^County #1 does not have information about participant age due to data collection errors, thus all observations are listed as “Unknown” in the table.

^b^Participants could select more than one option for race/ethnicity, so totals may exceed 100%.

^c^County #2 did not collect information on participants' education level due to partner organization preference, thus all observations are listed as “Unknown” in the table.

The majority of participants across groups were women (55%−100%), white (69%−100%), and between the ages of 31–60 (51%−75%). Most participants from Counties #1, #3, and #4 had at least a Bachelor's degree (43%−77%).

The following sections examine Photovoice participants' perspectives on the five core domains of the Centers for Medicare & Medicaid Accountable Health Communities Health-Related Social Needs categories (housing instability, food insecurity, transportation problems, utility help needs, and interpersonal safety) as well as the relevant structural determinants within these domains that participants discussed. Select photo-quote combinations developed by participants are also included to further illustrate the analytic findings.

### 3.1 The right to be housed—Entwining values and institutional practices

Photovoice groups in all four counties vocalized housing stability as a major health priority. In some instances shared by participants, lack of housing is a hindrance to the substance use disorder recovery process and even contributed to a young person's death. Elsewhere, the lack of government intervention and regulation makes neighborhoods unlivable when factories cause environmental hazards. Through the focus group discussions, participants highlighted how stigmatizing beliefs lead to cycles of discrimination and housing inequity in their communities, noting that “*there's a stigma just as bad on homelessness as there is addiction*.” The stigma against people experiencing homelessness results in these individuals being pushed to unlivable spaces at the margins of society. Despite stark, visible divisions between the “*haves and the have-nots*” where nice or “*luxury*” housing suddenly gave way to mobile home units or deteriorating apartment buildings, individuals and communities alike do not take the time to see and understand how the housing crisis came to exist. Some participants lamented the lack of dignity provided to people without housing in their community:

“*What kind of environment is [County #2] offering, to allow them to live houseless in a place… that's not covered in trash, that's not covered in… other people's belongings and their leftovers? What are we giving them as far as green space? What are we giving them as far as safety and security to make these decisions?”*

**Figure 1:**
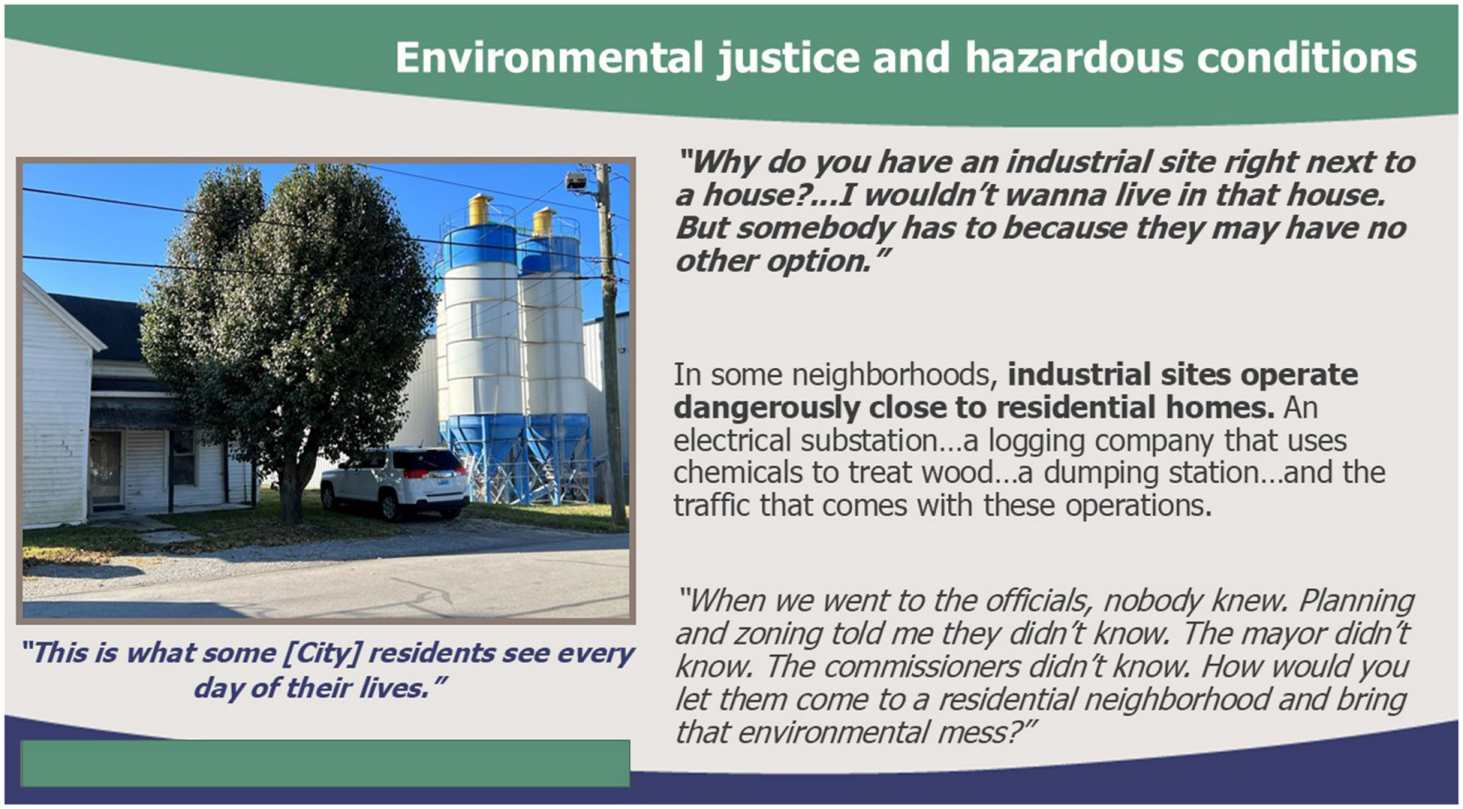
Photovoice photo-quote combination highlighting participant frustrations with hazardous environments.

One participant whose job responsibilities include connecting clients to housing explained that landlords refused to accept potential tenants who use vouchers from the Department of Housing and Urban Development, *despite the vouchers' ensuring that the landlords would receive rent money*. In other words, individuals with the power to decide who is housed may willingly forgo the opportunity for income to distance themselves from the stigma of government assistance. When government vouchers are accepted or housing is affordable, stigma against people without housing can become institutional practice through property neglect and lack of oversight—focus groups described affordable housing options as “*horrendous squat houses”, “ancient”*, or being in areas with high environmental and noise pollution due to the government's hands-off approach. One participant went so far as to name classism and racism directly as the root of their neighborhood's exposure to factory waste, and they felt snubbed by local leaders despite repeated advocacy efforts. Figure 1 demonstrates the lack of government intervention in providing environmentally safe residential areas. Thus, imbalanced power structures—such as those that value wealth and individual merit—become regular practice in housing policy through government inaction.

More often than not, affordable housing is virtually nonexistent or rapidly disappearing, and Photovoice discussions on gentrification further illustrated the connection between values and regulation and practice as contributors to the housing crisis. Participants criticized the stigma that people without housing have made bad decisions, pointing out that available jobs do not pay salaries that could afford the available housing options. New subdivisions sprouting in areas of need automatically disqualify individuals with low socioeconomic status before construction is even completed, and drastically increasing property values push residents out of neighborhoods with nowhere to go:

“*…you're pulling in people, you're not rehabbing the community that was already there. You're displacing that community that was there. Now what are you doing with them?”*

Even Photovoice participants with stable incomes shared their personal inability to find housing close to their workplaces because homes were unaffordable.

### 3.2 Who's responsible for feeding the community?

Photovoice participants did not call for individual responsibility to address food and nutritional concerns, but instead identified structural gaps that led to food insecurity in their communities. For example, one participant in County #1 brought up the need for healthy food options and implicated the lack of locations to obtain fresh foods rather than individual choices:

“*When we think about healthy food, we need to work on that. I know we have a lot of Dollar Stores on this end of town, but where are the healthy foods? Do we have to go to [Speaker 5] or to the churches just to get healthy food for the people that are walking on this end of town? …I know that we have the farmer's market, but I've only been there once… I know it's early in the morning on Saturday. I don't know if the hours need to be extended so people could get there and get healthy foods, but the food problem that we have, we need to work on people being able to get there.”*

**Figure 2:**
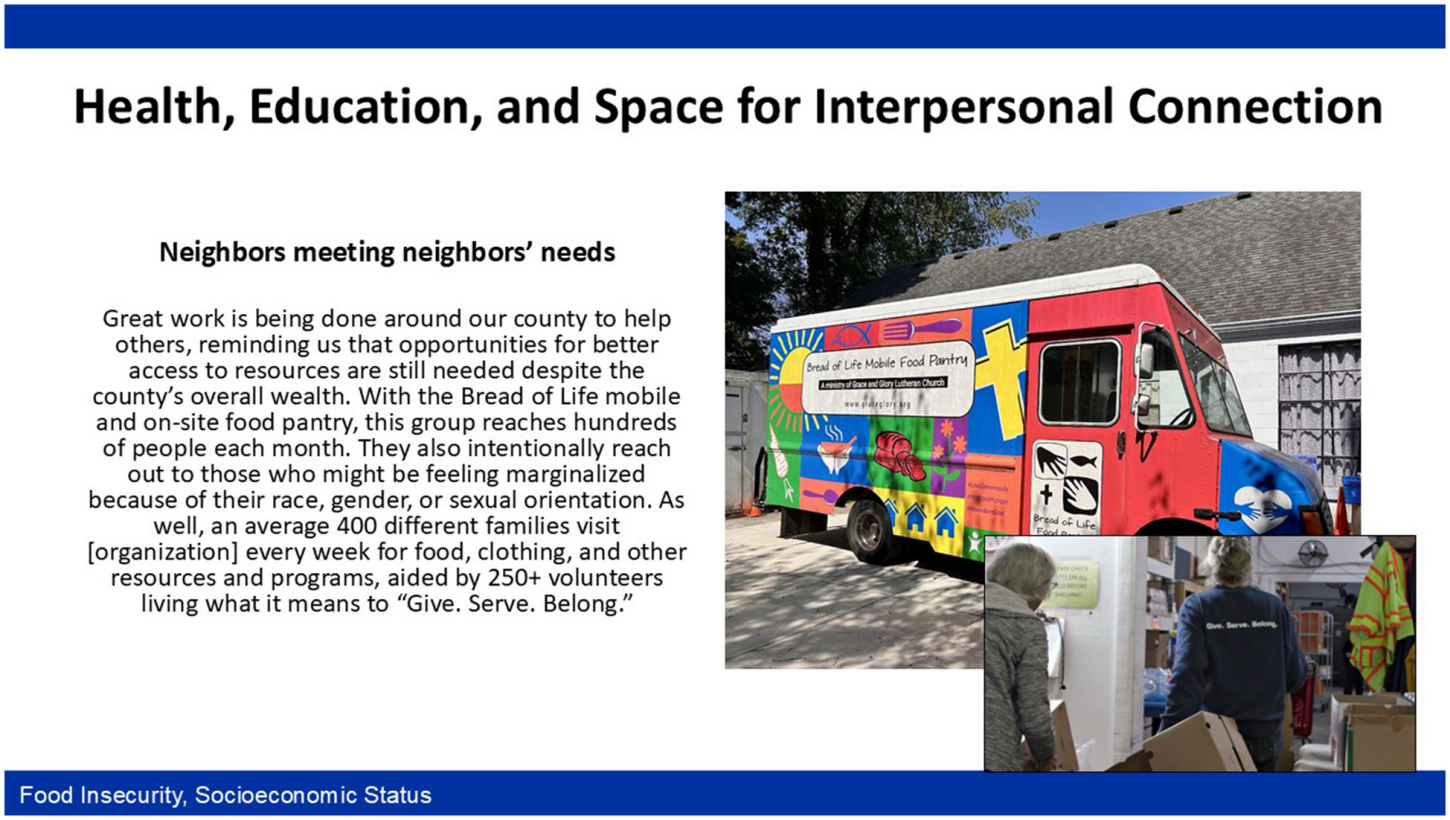
Photovoice photo-quote combination of local efforts to address food insecurity.

From the participant's perspective, a lack of nutritious food choices results from decisions about where people can access affordable food. In fact, focus group discussions highlighted the lengths people will go to meet this basic need. Participants in County #2 explained that the food pantry is first-come, first-served, and they had witnessed conflicts between individuals trying to cut in line. These examples highlight communities' reliance on non-governmental organizations such as churches and farmers' markets to fill gaps in government resources. In County #1, one church steps up to feed “*between 70 and 80 families per week*.” Figure 2 displays a photo-quote combination created by participants in County #4 that features local efforts to combat food insecurity.

So, even though government assistance may be available, it cannot meet the profound need, and individuals become dependent on the goodwill values of local organizations.

### 3.3 “Where the sidewalk ends”—Transportation as a fundamental cause

Transportation- and mobility-related infrastructure was a focus of much of the community health assessment group discussions. One participant tied the state of community transportation access to larger societal values:

“*…This is a larger societal issue, and it's an American thing probably in a lot of areas where we are a car society. …Even if we had bike lanes and pedestrian safe sidewalks, when we're in an oriented car-society, the motorists often don't even respect the rights of bikers or walkers. And so that would take a major shift in our cultural thinking to embrace walkability and any kind of transportation that wasn't actually an individual car…”*

In other words, the profound reliance on personally owned vehicles has been institutionalized through reduced governance of public transportation and community walkability.

In County #1, Photovoice participants felt that a lack of consistent transportation leads to reintegration struggles, as clients who finish residential treatment for substance use disorder cannot maintain employment when they cannot drive themselves to work. One participant mentioned a positive partnership between a provider and transport service that is meeting client needs, though more hours and route locations would benefit the community. Furthermore, medical crises become especially dire when understaffing of emergency medical transportation in rural areas leaves individuals vulnerable to severe illness or death, as one participant shared:

“*We have no emergency medical service in [City]. We have it, but it's so limited. So patients cannot obtain transport by EMS, or if we need to transport to a higher level of care, we have to use [Outside] County and we contract with [Outside] County to do transports to higher level of care or to nursing homes because 50% of the time, [City] Fire EMS is not available. They don't have the staff.”*

Participants in urban areas expressed similar issues about reliable transportation. While the participants who live in rural areas were frustrated at the logistics and time involved with booking medical transportation, participants in urban counties observed shortcomings with taking the bus. One participant from County #2 shared that substance use treatment clients are penalized for late attendance, even though providers know enough about their reliance on the bus system to offer passes for public transportation. Furthermore, Photovoice discussions detailed bus stops as a safety risk due to inadequate lighting, shelter, and seating. Despite community health leaders advocating for more stops and better conditions, funding and attention have not been directed to this health concern.

Related to discussions of inadequate transit, Photovoice participants across sites expressed frustration with the poor condition of sidewalks, which limited walkability. “*Where the sidewalk ends*” was a phrase used multiple times across groups, not as a metaphor, but as a literal description of areas with high foot traffic. One participant stated:

“*There is a notable lack of like pedestrian infrastructure. This is a major intersection in town… and there's not any kind of like crosswalk, no push button. There's no sidewalks, there's nothing. It's literally only for like vehicle traffic, which I think's a hindrance and unsafe, because people walk whether there's sidewalks or not. So, I think it's just a safety issue, safety concern.”*

Across all counties, Photovoice participants agreed with the above sentiment that the lack of sidewalks is a safety hazard because individuals who cannot access vehicle transportation must walk, regardless of walking conditions. A participant in County #2 alluded to the societal values around driving by pointing out that it is unfair to encourage people to walk and bike when there is little to no infrastructure to protect walkers and cyclists; the participant's frustration highlights the tension between calls for behavior change at the individual level and actions needed at the structural level. Additionally, city improvements to infrastructure have not met participants' expectations for enhancing safe walking conditions. For example, participants in County #4 assumed that recent improvements to an overpass would include a walking path and were disappointed when this was not the case. There was synergy with this sentiment in County #3, in which a participant felt like new businesses would “*just pop up*” with little planning around how people would reach those businesses. In County #1, participants saw the confusion about who shoulders the burden of fixing sidewalks leading to stagnation. For example, a participant believed the property owner is responsible for sidewalk maintenance and logically wondered whether the general public is aware of this or whether it makes sense to let sidewalks continue in disrepair if the property owner cannot afford the cost of maintenance. The discussion pushed back on the individual burden dictated by these nebulous regulations:

“*But it always comes down to the government. To me, they're supposedly in charge of keeping citizens safe. So I think, like you said, sidewalks like this aren't safe.”*

This participants' statement demonstrates the need for government and policy intervention—not action by individual community members—to promote the wellbeing of citizens.

### 3.4 Utility needs and the forces that leave rural communities behind

Photovoice discussions demonstrated the ways in which access to everyday utilities impacts community health. Internet access has become integral to the provision of health services since the onset of the COVID-19 pandemic, especially in rural communities that face provider shortages and long travel times to receive medical care. One participant shared that faulty internet access results in the failure to provide adequate mental healthcare to citizens, as psychiatrists and nurse practitioners struggle to maintain virtual contact with rural clients. This infrastructure concern is especially a crisis because Photovoice groups across all counties noted the impact of the opioid epidemic and the need for more resources in rural areas. While virtual healthcare options could be a way to address substance use issues in rural communities, the widespread inability to access reliable internet makes virtual healthcare unfeasible.

Although urban counties face challenges with internet access due to high costs, Photovoice participants from rural counties were frustrated by the absence of internet choices. As one participant who lives in an urban county but works in County #3 explained, there is no motivation for a company to provide sufficient services at a reasonable cost when there is no competition:

“*…If you only have one option for Internet services, then you only have one option. …Whatever they charge, that's what you have to pay if you want the Internet. There's no competition, like where I live… I could have [Company #1], or I could have [Company #2], or I could have [Company #3], there's different options. And you can switch… because you have the choices. But here… some people can't get Internet at all. If you only have [Company #1], then even if it's available, you might not be able to afford it.”*

Thus, regional monopolies on internet access leave rural communities further isolated and distanced from the resources that could improve their health outcomes. Government intervention was the solution proposed by participants to address high costs and limited access. A participant from County #1 summarized the need for structural change:

“*I would be an advocate for free internet service because it is no longer a luxury. It's a necessity. And I believe in the expansion of broadband. And also then a way to make this service available to everybody.”*

In assessing community health, Photovoice participants yet again highlighted an interplay of values and regulation that set health promotion beyond the scope of local health departments doing their best to serve the community.

### 3.5 Structural considerations related to interpersonal safety

Photovoice discussions related to interpersonal safety evoked a sense of individual helplessness against the threat of violence, largely due to systemic conditions. This structural vulnerability was apparent as participants shared fears of experiencing mass gun violence. The onus of gun violence prevention was placed on government regulation and policy, and Photovoice participants perceived legislative bodies as failing to prioritize their wellbeing. For example, one participant stated:

“*And where was it… Decided they took hard action. We haven't. And then as a result, you can see the difference in that [gun violence]. This is still continued for us.”*

Participants also noted that prevention efforts have been stymied by a lack of government funding at the federal for firearm violence research, again pointing to the ways in which policy and practice have shaped public safety and normalized mass shootings. In place of institutional protections, organizations are left to develop their own procedures. This was demonstrated by a participant's thoughts on the aftermath of the Virginia Tech shooting:

“*And so we were all scrambling as college administrators and directors to figure out, okay, how are we going to respond should this happen to us? …And back then, we didn't have a one-call system. We didn't have the mass texting capabilities that we all do now. And so we had to really come up with a phone tree, was really the only thing we can do. And now, these mass texting and these apps and stuff, some of them came about because of mass shootings.”*

The excerpt illustrates how organizations, such as campuses, step up to promote safety when there are no guiding policies.

Interpersonal safety also emerged from Photovoice discussions about participants balancing safety risks while doing their jobs. The following excerpt shows the complicated feelings of working in public health:

“*And so I think many of us are very understanding and compassionate to like what's going on here in this downtown area. But then, there are these concerns of, maybe I need to lock my door. Maybe I need to be a little bit more vigilant.”*

The quote highlights that while participants who work in service provision recognized the importance of their work, they struggled with feelings of vulnerability, knowing that clients' unstable circumstances can result in conflicts. In some cases, on-site security helped Photovoice participants feel safer at their workplaces, but the availability of security staff was contingent on funding and decisions that the cost of extra staff was worth the reduction in potential risk to employees. Thus, interpersonal safety becomes contingent upon institutional budgeting priorities.

Finally, interpersonal safety was also threaded through focus group discussions of belonging, as participants expressed concerns of isolation due to marginalized identities. For example, one participant in County #4 shared “the nerves [they] had… walking around town with a Pride flag” hoping to be the representation they feel is lacking in their county. Based on the focus group discussion, the connection between values and practice can be made through institutional decisions about who is allowed to use which restrooms, a large part of the current local discourse. Therefore, in a similar way to the other themes, interpersonal safety in its various forms is an undertaking beyond local health departments' capacity to sufficiently address within their scopes and resources.

## 4 Discussion

This study revealed how community members in rural and urban Kentucky counties experience the structural determinants of health as localized challenges with housing instability, food insecurity, transportation, Internet access, and interpersonal safety. Participants attributed responsibility for inequitably distributed health-related social needs in their communities to the interplay of structural determinants like values and regulations of local government decision-makers, rather than the individual behavior of community members. Focus group discussions revolved around the relationships among stigmatizing attitudes, beliefs, and practices toward people who utilize public services and local government inaction in responding to health-related social needs. For example, several participants described a cycle of discrimination and housing inequity driven by stigmatizing beliefs that legitimize property neglect, lack of oversight, and high exposure to environmental and noise pollution for publicly subsidized housing, with gentrification displacing lower-income people from newly constructed housing. Similarly, rather than blaming individual behaviors, participants ascribed food insecurity to the systemic inadequacy of current publicly funded nutrition resources and overreliance on charitable organizations in meeting their communities' basic food needs. The effort participants have seen citizens exerting to obtain food conflicts with the idea that nutrition can be resolved through health initiatives that prioritize individual behaviors, and so food systems need to be critically examined at the policy level to address food insecurity.

Participants also viewed limited transportation options for accessing health-related resources to be the result of local policy decisions that privilege private transportation at the expense of public options. Several participants described the predominance of private transportation as the assumed norm for local policymaking, which undercuts efforts to improve walkability and pedestrian safety. Rural areas face distinct transportation limitations, including limited public transportation and longer travel distances (22), which is supported by participant conversations related to unreliable or nonexistent bus routes. Existing supports like Medicaid transportation alleviate some transportation challenges but require advanced notice, so last-minute schedule changes may be difficult to navigate. Telehealth should offer information technology solutions to these rural transportation and healthcare access and provider shortage issues, but participants cited unreliable, high-cost Internet services resulting from monopolistic carrier practices.

Finally, participants explained how the lack of policy solutions, funding, and political will to regulate firearms and protect front-line workers and marginalized identities are fundamental causes of interpersonal violence. Despite the willingness of local organizations to implement safety mechanisms, their responses can only be reactive; the mass texting chains described by one participant above can only take place once a shooting has begun. Without legislation to designate funding for preventive measures against firearm violence, mass shootings will continue to threaten community safety. The conversation about firearm violence prevention illustrates a larger interpersonal safety concern in which the burden of safety is placed on individual and local organizations rather than political institutions–this was perceived as the case regardless of conversations about worker safety, acceptance of marginalized identities, or prevention of firearm violence.

These findings not only have important implications for local- and state-level public health practice but also underscore the value of participatory action research methods like Photovoice in visualizing community members' conceptualizations of how social and structural determinants impact population health outcomes. These Photovoice projects integrated into local health department CHA/CHIP practice showed the significance of fundamental causes in undermining traditional behavior change-based public health approaches. They also align with previous CHA Photovoice data reflecting participants' desire to shift unresponsive local government dynamics toward resolving health-related social needs (6). Addressing fundamental causes typically requires the political will and economic power of local politicians and developers that local health departments cannot marshal in isolation, given their mission, scope, and funding. The insufficient local and state government intervention in their communities' response to social and structural determinants detailed by study participants highlights the imperative to advance health equity through the political determinants of health—voting, government, and policy—that systematically administer power, structure relationships, and distribute resources (23).

Thus, this study's findings amplify calls to realize the Public Health 3.0 model for improving population health (15, 24). The Public Health 3.0 initiative envisions local health departments serving as chief health strategists who mobilize cross-sector resources to address their communities' upstream social determinants of health for collective impact (15, 24). We conclude that Public Health 3.0-style mobilization of structured cross-sector partnerships in shared funding, services, governance, and collective action is critical to promoting local- and state-level investment in affordable housing and nutritious food, public transportation, Internet utilities, and safe environments. Public Health 3.0 can also promote policies to curb the harms of unmitigated private development and corporate profiteering. These partnerships can set shared priorities based on Photovoice and other actionable data to guide funding braiding and blending, as well as capture cost savings for long-term reinvestment in public housing and transportation, sustainable food and informational technology systems, and public safety (15). In particular, local health department coordination with non-profit hospitals to inform and document the contributions of their required community health needs assessments and community benefit activities could powerfully influence public health systems investments and policies addressing social, structural, and political determinants (25–31).

Additionally, this study corroborates previous findings (6, 32) that Photovoice is an accessible and feasible way for local health departments to meet community members where they are, gather insightful qualitative data for CHAs, and identify community health concerns that may be difficult to draw out through traditional survey techniques. These Photovoice projects exemplify the method's promise to move participants from passive adaptation to their communities' conditions to emotional and cognitive engagement with these issues, leading to intention to take action to address structural and political determinants and health-related social needs (1, 33, 34). The consciousness-raising qualities of Photovoice photographs and visual elements also create readily shareable products that are likely to be more impactful to other community members, community leaders, and policymakers than statistics alone. Furthermore, integration of Photovoice into the local health department CHA/CHIP process can help address critiques that the method typically fails to accomplish its original aim of reaching policy- and decision-makers to advocate for adoption of health-promoting policies and actions (35–38).

For the Photovoice projects included in this study, the goal of the final session—referred to as the analysis and action planning session—was to organize participant priorities and materials into products for dissemination. Each group collaboratively selected a few key strengths photos and barriers photos that they agreed best represented their group's main takeaways, and participants used the session time to develop photo captions that contextualized the photos with group consensus on priority health needs. Photo-quote combinations were compiled into posters that were shared at community health forums as part of the local CHA process attended by local decision-makers and community members; the posters were publicly displayed at the health department, or in the case of County #3, at the local library. As part of local health department CHAs, the project findings were included in the to inform the policies, strategies, and interventions prioritized by the respective health departments during the community health improvement planning (CHIP) phase. Thus, participants were able to have a voice in and contribute to their county-level governance pertaining to addressing local health needs.

Beyond local-level CHAs, this study points to the potential impact of conducting larger-scale comparisons of Photovoice projects to assess similarities and differences in residents' lived experiences of health-related social needs across U.S. communities, states, and regions and in relation to other local-level data. Comparative analyses also can help overcome the limitations to generalizability of small, community-specific sample sizes. Additionally, longitudinal comparisons of Photovoice data could support longer-term cycles of strategic planning and accountability in addressing structurally determined health-related social needs (6, 39). For example, implementation of Photovoice during subsequent CHA/CHIP cycles of the counties included in the present study would provide insight into whether community priorities have been sufficiently addressed by local health initiatives, or indicate reasons why health barriers persist over time despite local strategizing. Because our study participants were recruited through local health departments and therefore typically were already engaged in community health improvement efforts in some capacity, the incorporation of Photovoice projects into CHA/CHIP practice could entail purposive sampling and recruiting in partnership with community members and organizations most directly affected by structurally determined social and health needs. Photovoice also could be utilized in a State Health Assessment as qualitative data to inform community perceptions of the built and social environment, thereby strengthening both CHA and ability to meet standards and measures for reaccreditation by the Public Health Accreditation Board.

This study has some limitations to be noted. First, Photovoice participants were recruited via convenience sampling by health department liaisons. Because of the recruitment strategy, participants were likely highly motivated and engaged in community health promotion prior to the study, and so they may not be representative of the counties in which they resided. Future CHA implementation efforts could address this by using broader recruitment strategies and engaging with multiple local organizations instead of solely health departments. Another limitation is the lack of control over the demographic data obtained from participants, which resulted in demographic data missing from Table 1. However, the discussion transcripts were rich with contextualization of health issues within the counties included in the study, and repeating the Photovoice in future CHA efforts with more targeted sampling strategies can provide longitudinal data on the persistent gaps in local health improvement efforts.

## 5 Conclusion

Structural and political determinants of health are fundamental causes of health disparities but are difficult to assess and address by governmental local health departments alone without cross-sector buy-in and collaboration. Embedded in a participatory action research paradigm but without requiring participants to disclose their identities, Photovoice can be an impactful method for grounding community- and state-level public health planning efforts in lived experiences of structural and social determinants of health. Photovoice data can thus contribute rich contextual understanding of often-stigmatized health-related social needs to public health research and practice.

## Data Availability

The deidentified data supporting the conclusions of this article will be made available by the authors, without undue reservation.
